# Spermidine alleviates cardiac aging by improving mitochondrial biogenesis and function

**DOI:** 10.18632/aging.102647

**Published:** 2020-01-06

**Authors:** Junying Wang, Shaoqi Li, Ju Wang, Feixiang Wu, Yuhan Chen, Hao Zhang, Yubo Guo, Yan Lin, Lingxu Li, Xue Yu, Ting Liu, Yajun Zhao

**Affiliations:** 1 Department of Pathophysiology, Harbin Medical University, Harbin, China; 2 Department of Medical Technology, Beijing Health Vocational College, Beijing, China; 3 Affiliated Hospital of Hebei University, Baoding, China; 4 Department of Pathology, The First Affiliated Hospital of Soochow University, Suzhou, China; 5 The Second Affiliated Hospital of Harbin Medical University, Harbin, China; 6 Department of Pathophysiology, Qiqihar Medical University, Qiqihar, Heilongjiang, China; 7 Key Laboratory of Cardiovascular Medicine Research, Harbin Medical University, Ministry of Education, Harbin, China

**Keywords:** spermidine, polyamine metabolism, SIRT1, PGC-1α, mitochondrial biogenesis

## Abstract

Polyamines have been shown to delay cellular and organismal aging and to provide cardiovascular protection in humans. Because age-related cardiovascular dysfunction is often accompanied by impaired mitochondrial biogenesis and function, we explored the ability of spermidine (SPD), a major mammalian polyamine, to attenuate cardiac aging through activation of mitochondrial biogenesis. Cardiac polyamine levels were reduced in aged (24-month-old) rats. Six-week SPD supplementation restored cardiac polyamine content, preserved myocardial ultrastructure, and inhibited mitochondrial dysfunction. Immunoblotting showed that ornithine decarboxylase (ODC) and SPD/spermine N1-acetyltransferase (SSAT) were downregulated and upregulated, respectively, in the myocardium of older rats. These changes were paralleled by age-dependent downregulation of components of the sirtuin-1/peroxisome proliferator-activated receptor gamma coactivator alpha (SIRT1/PGC-1α) signaling pathway, an important regulator of mitochondrial biogenesis. SPD administration increased SIRT1, PGC-1α, nuclear respiratory factors 1 and 2 (NRF1, NRF2), and mitochondrial transcription factor A (TFAM) expression; decreased ROS production; and improved OXPHOS performance in senescent (H_2_O_2_-treated) cardiomyocytes. Inhibition of polyamine biosynthesis or SIRT1 activity abolished these effects. PGC-1α knockdown experiments confirmed that SPD activated mitochondrial biogenesis through SIRT1-mediated deacetylation of PGC-1α. These data provide new insight into the antiaging effects of SPD, and suggest potential applicability to protect against deterioration of cardiac function with aging.

## INTRODUCTION

Mitochondrial dysfunction is considered a major contributor to aging, a dominant risk factor for the development of cardiovascular disease [1, 2]. Recently, cell senescence-related defects in the generation of new mitochondria (mitochondrial biogenesis) have been the focus of intensive research [3]. Peroxisome proliferator-activated receptor γ coactivator-1 α (PGC-1α) is a master regulator of mitochondrial biogenesis. PGC-1α enhances the activities of nuclear respiratory factor 1 and 2 (NRF1, NRF2), which induce the transactivation of several genes encoding mitochondria-specific proteins involved in the respiratory chain, mitochondrial DNA (mtDNA) transcription/replication, and protein import/assembly [4]. NRF-1 regulates the expression of mitochondrial transcription factor A (TFAM), a key activator for the replication, transcription, and stabilization of mtDNA [5], which promotes the expression of mitochondria-related proteins and contributes to mitochondrial biogenesis. Cardiac-specific deletion of NRF-1 and TFAM is associated with decreased mitochondrial content or function [6, 7]. Dysregulation of PGC-1 α expression has been observed in cardiac hypertrophy, heart failure, chronic cardiomyopathy, and other cardiac pathologies [8–12]. PGC-1α protein levels were found to be reduced in aged mice, and loss of PGC-1α has been suggested to be an important contributor to mitochondrial dysfunction in aging-associated diseases [13, 14].

Mammalian sirtuin 1 (SIRT1) is a nicotinamide adenine dinucleotide (NAD)-dependent deacetylase involved in a wide range of physiological and pathological processes [15, 16]. SIRT1 promotes mitochondrial function in response to fasting by deacetylating PGC-1α in skeletal muscle [17]. Upregulation of Sirt1 and PGC-1α is often linked to increased life span [18]. Systemic deletion of SIRT1 in mice induces development of dilated cardiomyopathy accompanied by mitochondrial dysfunction [19]. SIRT1 affords a protective role against myocardial ischemic/reperfusion injury and has been shown to negatively regulate oxidative stress, a condition that contributes to cardiac aging [20, 21]. In mice, SIRT1 was further shown to protect the aging heart from contractile dysfunction through inhibition of cellular apoptosis [18]. Notably, SIRT1 activation can promote mitochondrial biogenesis and function in cardiomyocytes through its deacetylating effect on PGC-1α, upregulating in turn the expression of NRF1, NRF2, and TFAM in a diabetic cardiomyopathy mouse model [22].

Mammalian polyamines include putrescine (PUT), spermidine (SPD), and spermine (SPM). Polyamines are derived from amino acid catabolism and are present in almost all eukaryotic and prokaryotic cells [23]. Many functions have been ascribed to polyamines, including regulation of ion channels, DNA and RNA stability, inhibition of inflammation, regulation of DNA methylases, protein acetylation, and stress resistance [24, 25]. Ornithine decarboxylase (ODC) and spermidine/spermine N1-acetyltransferase (SSAT) are key enzymes of polyamine synthesis and catabolism, respectively. It has been shown that intracellular polyamine levels decrease with age in various species [26, 27], and genetically-induced depletion of intracellular polyamines decreases life span in yeast and mice [28, 29]. Conversely, SPD supplementation prolonged life span and reduced age-related pathology [30]. In various biological models, SPD was shown to increase heat, hydrogen peroxide, and paraquat stress resistance, and decrease age-related oxidative damage [31, 32]. Accumulating evidence indicates that SPD’s beneficial effects on aging are mainly due to the induction of autophagy [33].

Recent studies highlight the benefits of polyamines for the cardiovascular system [34, 35]. SPD administration reduced lipid accumulation and necrotic core formation by inducing autophagy in an atherosclerosis mouse model [34]. Polyamine exposure attenuated cardiac endoplasmic reticulum stress during acute myocardial infarction by inhibiting reactive oxygen species (ROS) production in isolated, perfused rat hearts [36]. Interestingly, a large multi-center work showed that exogenous SPD administration enhanced mitophagy, promoted mitochondrial respiration, and improved diastolic function to delay cardiac aging in mice. In addition, the study revealed that high levels of dietary SPD were inversely correlated with cardiovascular disease in humans [35]. Our previous studies suggested that exogenous polyamines protect against myocardial reperfusion injury by inhibiting mitochondrial permeability transition pore (mPTP) opening [37], and provided novel information derived from combined proteomics and metabolomics analyses on the cardioprotective effects of polyamines in the aging heart [38]. To address important knowledge gaps that remain in relation to the role of polyamines in cardiomyocyte aging and function, the present work examined the effect of exogenous polyamine administration on mitochondrial biogenesis and function in the aging heart.

## RESULTS

### Exogenous spermidine supplementation restores polyamine metabolism and attenuates cardiac aging

**Figure 1:**
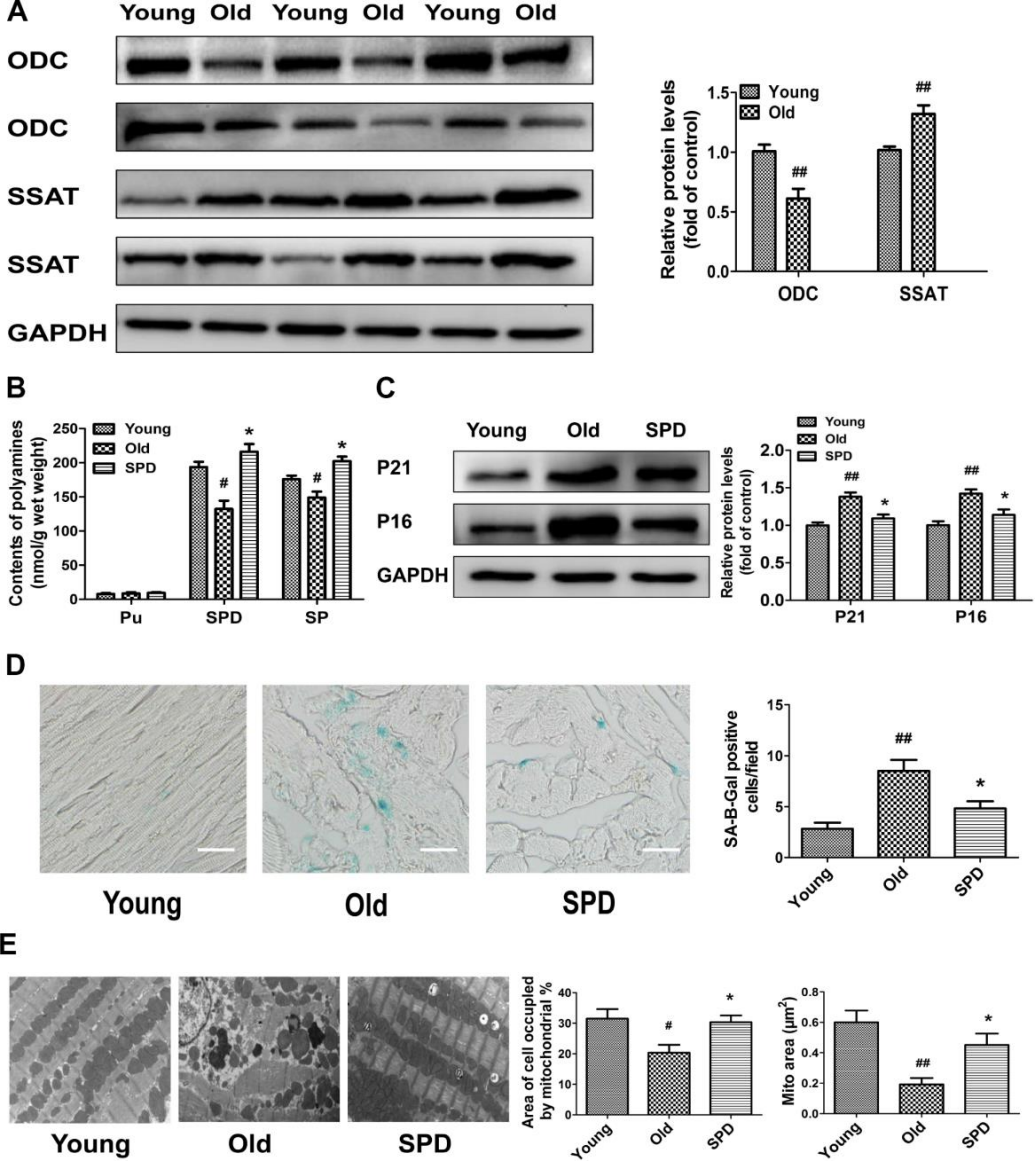
**Age-dependent changes in polyamine metabolism and effect of spermidine on cardiac aging in rats.** (**A**) Representative immunoblot bands for ODC and SSAT and quantification of protein levels in the myocardium of 3-month-old (Young) and 24-month-old (Old) rats. GAPDH was used as loading control. (**B**) Polyamine content, including putrescine (PU), spermidine (SPD), and spermine (SP) in the myocardium of young, old, and SPD-treated old rats (SPD group) evaluated by high-performance liquid chromatography. (**C**) Representative immunoblot bands for p21 and p16 and quantification of protein levels. GAPDH was used as loading control. (**D**) Positive area of senescence-associated β-galactosidase (SA-β-gal) staining. Scale bars: 20 μm. (**E**) Representative transmission electron microscopy images showing ultrastructural changes in the myocardia. Quantification of the area of cells occupied by mitochondria (%); Magnification: ×10,000. n = 6 for each group. ^#^ P < 0.05 vs. young control, ^##^ P < 0.01 vs. young control, ^*^ P < 0.05 vs. old.

To study polyamine metabolism in the myocardium of 3-month-old (young) and 24-month-old (old) rats, we assessed ODC and SSAT protein expression, as well as polyamine contents. The expression of ODC decreased, while the expression of SSAT increased, in older rats compared with younger ones (Figure 1A). Meanwhile, both spermine (SPM) and spermidine (SPD) contents were significantly reduced in the myocardium of older animals, which showed significant elevations in both polyamines after 6-week treatment with SPD (Figure 1B). To evaluate the potential anti-aging effects of SPD on myocardial tissue, three age-related parameters were assessed: 1) senescence-associated-β-galactosidase (SA-β-gal) activity, 2) accumulation of p21 and p16 proteins, and 3) changes in the ultrastructure of myocardium. We found that SA-β-gal staining and p21/p16 expression was increased in the hearts of older rats, and these effects were attenuated by SPD supplementation (Figure 1C and 1D). Abnormalities in the left ventricular ultrastructure, including sarcomere deformation, lipofuscin deposition, decreased mitochondrial matrix density, mitochondrial cristae disorganization, and inner mitochondrial membrane damage, were also evident in aged rats after electron microscopy evaluation. However, following SPD treatment, sarcomere structure was clear, and the mitochondria were tightly packed between the myofibrils and had intact outer and inner membranes with distinct cristae (Figure 1E). These data showed that in aging hearts SPM and SPD contents are reduced, denoting impaired polyamine metabolism, whereas SPD supplementation markedly protected against heart senescence through replenishment of the cardiac total polyamine pool.

### Exogenous spermidine supplementation protects against H_2_O_2_-induced cardiomyocyte aging

**Figure 2:**
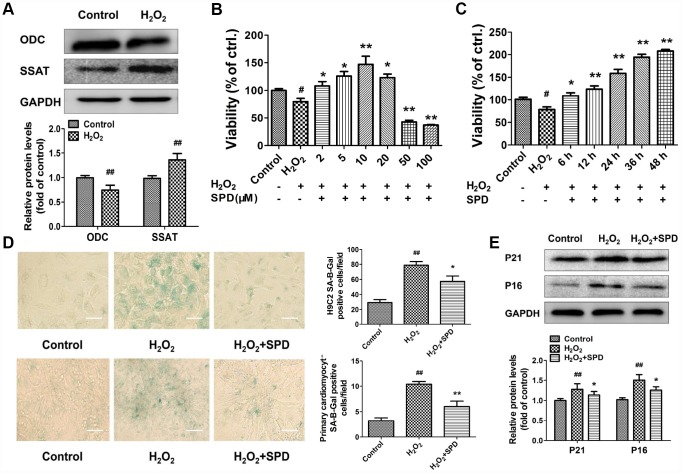
**Expression changes in ODC and SSAT and effect of SPD in H_2_O_2-_ treated cardiomyocytes.** (**A**) Western blot analysis of ODC and SSAT expression in NRCMs treated with or without 40 μM H_2_O_2_. GAPDH was used as loading control (n = 4). (**B**, **C**) Concentration- and time-dependent effect of SPD on the viability of NRCMs treated with 40 μM H_2_O_2_ (n = 8). (**D**) SA-β-gal staining in NRCMs (top) and H9C2 cells (bottom). The positive area of SA-β-gal staining is shown in the right-hand graph (n = 8). Scale bars: 20 μm. (**E**) Representative immunoblot bands for p21 and p16, and quantification of protein levels in NRCMs (n = 4). ^#^ P < 0.05 vs. control, ^##^ P < 0.01 vs. control, ^*^ P < 0.05 vs. H_2_O_2_, ^**^ P < 0.01 vs. H_2_O_2_.

To evaluate the effect of SPD on cardiomyocyte aging, we induced senescence in primary neonatal rat cardiomyocytes (NRCMs) and H9C2 myoblasts by treatment with 40 μmol/L H_2_O_2_ for 4 h (see Supplementary Material for optimization protocol). After H_2_O_2_ treatment, the expression of ODC decreased, and the expression of SSAT increased significantly compared with control cells (Figure 2A). Next, senescent NRCMs were treated with different doses of SPD (0–100 μmol/L) for 48 h. Results showed that SPD increased cell viability in a dose-dependent manner (Figure 2B). However, a decrease in cell viability was observed with SPD concentrations ≥20 μmol/L. We verified that after treatment with 40 μmol/L H_2_O_2_, supplementation with 10 μmol/L SPD for 6, 12, 24, 36, and 48 h increased cell viability in a time-dependent manner (Figure 2C). We further observed that the SA-β-gal–positive staining area in both NRCMs and H9C2 cells (Figure 2D), as well as p21 and p16 expression in NRCMs (Figure 2E), were significantly increased after senescence induction with H_2_O_2_; these changes were in turn prevented by supplementation with 10 μmol/L SPD. These data indicated that an imbalance in polyamine metabolism occurs in cardiomyocytes after H_2_O_2_-induced aging, and this could be ameliorated by exogenous SPD supplementation.

### Spermidine attenuates cardiac senescence via inhibiting ROS accumulation and improving mitochondrial function

**Figure 3:**
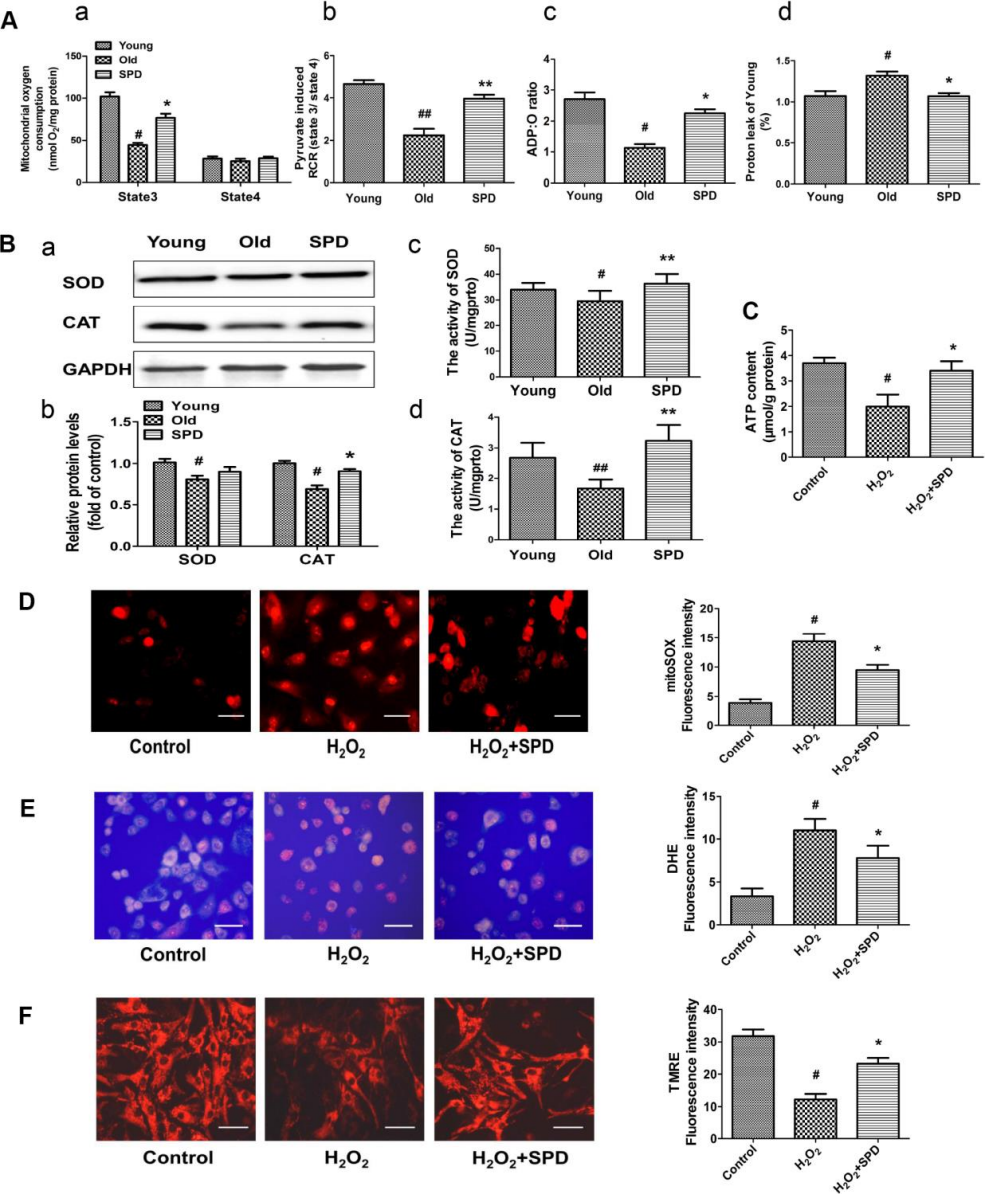
**Effect of SPD on mitochondrial respiration and ROS accumulation in the aged heart and in H_2_O_2_-treated cardiomyocytes.** (**A**) Mitochondrial oxidative phosphorylation (OXPHOS) efficiency was evaluated in the rat myocardium. Measurements included mitochondrial oxygen consumption States 3 and 4 (a), respiratory control rate (RCR) (b), P/O ratio (c), and proton leakage (d). Respiration was induced with pyruvate/malate (5 mM each) as energizing substrates and ADP (200 μM) to initiate State 3 respiration (n = 8). (**B**) Western blot analysis of SOD and CAT expression (a, b), and colorimetric detection SOD and CAT activity (c, d). n = 4 for protein expression and n = 8 for activity assay. ^#^ P < 0.05 vs. young control, ^##^ P < 0.01 vs. young control; ^*^ P < 0.05 vs. old, ^**^ P < 0.01 vs. old. (**C**) ATP content of cardiomyocytes measured by luminometry in NRCMs (n = 8). (**D**) Superoxide production in mitochondria detected by MitoSOX staining in H9C2 cells. (**E**) ROS production in H9C2 cells detected by DHE in H9C2 cells. (**F**) Mitochondrial transmembrane potential (ΔΨ*m*) detected by TMRE in H9C2 cells. Quantification of the mean fluorescence intensity of MitoSOX, DHE, and TMRE are displayed on the right side of the graphs (n = 8). ^#^ P < 0.05 vs. Control, ^##^ P < 0.01 vs. Control, ^*^ P < 0.05 vs. H_2_O_2_ group, ^**^ P < 0.01 vs. H_2_O_2_ group.

To confirm the mitochondrial-protective action of SPD, myocardial mitochondrial oxygen consumption and anti-oxidative ability were measured in isolated mitochondria after SPD administration in vivo. Compared with the young heart, the aged myocardium showed decreases in mitochondrial State 3 respiration, respiratory control ratio (RCR), and P/O ratio, and an increase in proton leakage, whereas exogenous SPD supplementation reversed these defects (Figure 3A, a–d). In addition, SPD supplementation reversed the decrease in superoxide dismutase (SOD) and catalase (CAT) expression and activity observed in the aging myocardium (Figure 3B a–d). Mitochondria are responsible for producing ATP, the main cellular energy molecule, in a process that also generates small amounts of reactive oxygen species (ROS). Therefore, ATP and ROS production, and mitochondrial membrane integrity are important indicators of mitochondrial function. We found that ATP levels decreased in H_2_O_2_-treated NRMCs, and SPD supplementation inhibited such decrease (Figure 3C). In addition, SPD treatment attenuated oxidative stress in H_2_O_2_-treated H9C2 cells, evidenced by a significant reduction in the fluorescence intensity of the mitochondria-specific superoxide indicator triphenylphosphonium-linked hydroethidium (mitoSOX) (Figure 3D) and the intracellular superoxide indicator dihydroethidium (DHE) (Figure 3E). Mitochondrial transmembrane potential (ΔΨm) was next monitored by the potentiometric dye tetramethylrhodamine ethyl ester (TMRE). In H9C2 cells, ΔΨm was significantly decreased by H_2_O_2_; however, after addition of SPD, ΔΨm remained intact (Figure 3F). In summary, these findings indicate that SPD alleviates aging-associated mitochondrial damage in vivo and vitro through inhibiting oxidative stress.

### Spermidine stimulates mitochondrial biogenesis and function via activation of the SIRT1/PGC-1α signaling pathway

**Figure 4:**
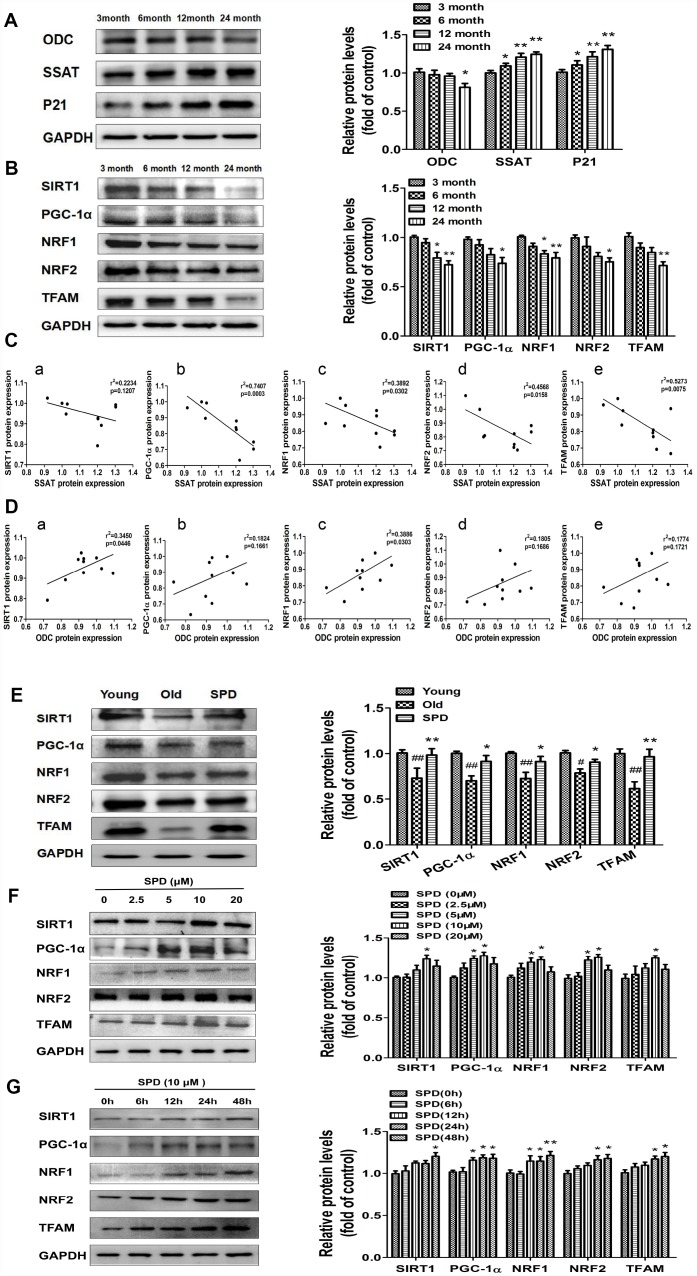
**SPD prevents age-associated depletion in myocardial polyamines and alterations of SIRT1/PGC-1α signaling pathway proteins.** Representative immunoblot bands for ODC, SSAT, and p21 (**A**), and for SIRT1, PGC-1α, NRF1, NRF2, and TFAM (**B**) in myocardium from 3-, 6-, 12- and 24-month-old rats. GAPDH was used as loading control. (n = 10). ^*^ P < 0.05 vs. 3 months, ^**^ P < 0.01 vs. 3 months. (**C**) Correlation between ODC and (a) SIRT1, (b) PGC-1α, (c) NRF1, (d) NRF2, and (e) TFAM in cardiac tissue from rats of different ages. (**D**) Correlation between SSAT and (a) SIRT1, (b) PGC-1α, (c) NRF1, (d) NRF2, and (e) TFAM in cardiac tissue from rats of different ages (n = 10). (**E**) Representative immunoblot bands for SIRT1, PGC-1α, NRF1, NRF2, and TFAM in the myocardium of young (3 months of age), old (24 months of age), and SPD-treated (6 weeks) old rats. (n = 4). ^#^ P < 0.05 vs. young, ^##^ P < 0.01 vs. young, ^*^ P < 0.05 vs. old, ^**^ P < 0.01 vs. old. (**F**, **G**) Expression of SIRT1, PGC-1α, NRF1, NRF2, and TFAM measured by western blot in NRMCs treated with 0, 2.5, 5, 10, or 20 μM SPD for 24 h (**F**) or 10 μM SPD for 0, 6, 12, 24, and 48 h (**G**). Quantification of protein expression is shown on the right-hand side of the graphs (n = 4). ^*^ P < 0.05 vs. control, ^**^ P < 0.01 vs. control.

To evaluate potential correlations between polyamine metabolism and the SIRT1/PGC-1α signaling pathway, a key regulator of mitochondrial biogenesis, western blot was used to detect the expression of ODC, SSAT, p21, and members of the SIRT1/PGC-1α signaling pathway in cardiac tissue of rats aged 3, 6, 12, and 24 months. We found that the expression of SIRT1, PGC-1α, NRF1, NRF2, and TFAM decreased depending on age (Figure 4B). Age-dependent changes seemed also apparent for ODC, SSAT, and p21 expression, i.e. ODC decreased, while SSAT and p21 increased with age (Figure 4A). A strong negative correlation was observed between SSAT and PGC-1α, NRF1, NRF2, and TFAM expression (Figure 4C). In turn, a significantly positive correlation was seen between ODC and both SIRT1 and NRF1 (Figure 4D). Meanwhile, SPD administration increased cardiac expression of SIRT1 and other mitochondrial biosynthesis-related proteins in the oldest rats (Figure 4E).

In vitro studies were also performed using NRCMs. We treated NRCMs with various doses of SPD (0-20 μmol/L) for 24 h after senescence induction with H_2_O_2_. SPD exposure (5 and 10 μmol/L) significantly increased the expression of PGC-1α*,* NRF1, and NRF2, while 10 μmol/L SPD increased the expression of SIRT1 and TFAM (Figure 4F). Using a fixed dose of 10 μmol/L SPD, we further observed that the expression of these proteins increased in a time-dependent manner (Figure 4G). These data suggest that SPD might delay aging of cardiomyocytes by activating the SIRT1/PGC-1α signaling pathway.

**Figure 5:**
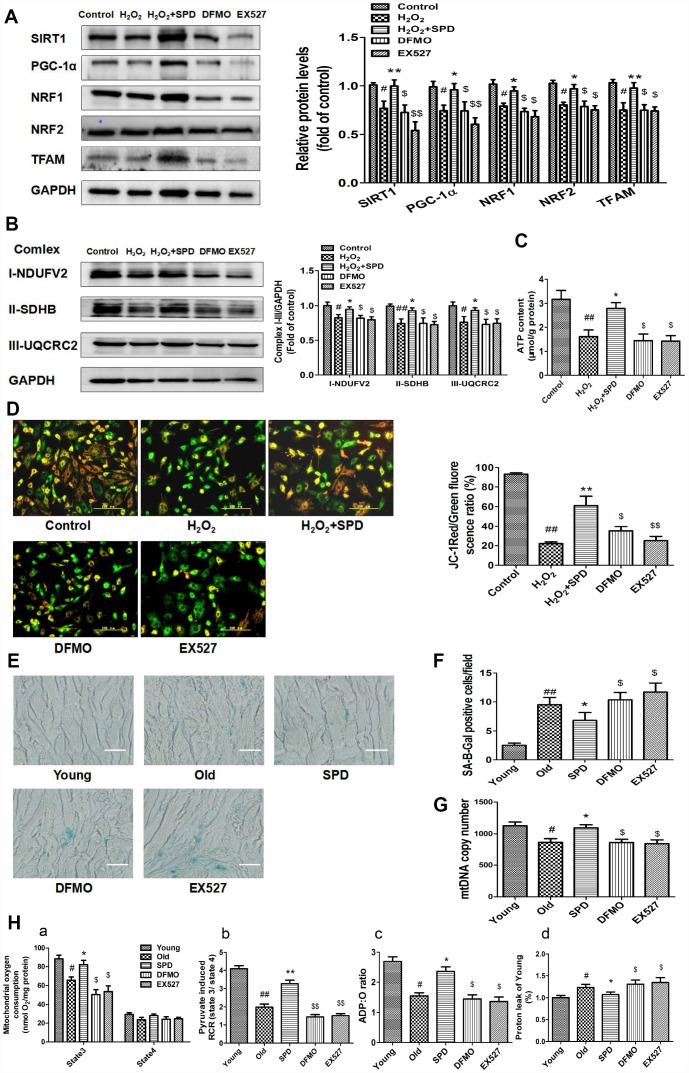
**Inhibition of polyamine biogenesis and SIRT1 activity attenuates SPD-induced mitochondrial biogenesis and functional improvement in aging cardiomyocytes.** For in vitro studies, NRMCs and H9C2 cells were cultured as follows: normal culture (Control), H_2_O_2_ treatment-induced aging (H_2_O_2_), H_2_O_2_ plus SPD (H_2_O_2_ + SPD), H_2_O_2_ plus SPD and DFMO (DFMO), or H_2_O_2_ plus SPD and EX527 (EX527). (**A**) Representative immunoblot bands for SIRT1, PGC-1α, NRF1, NRF2, and TFAM, and quantification of protein expression in NRMCs. GAPDH was used as loading control (n = 4). (**B**) Representative immunoblot bands for OXPHOS complexes I (NDUFV2), II (SDHB), and III (UQCRC2), and quantification of protein expression in NRMCs (n = 4). (**C**) ATP content measured by luminometry in NRMCs (n = 8). (**D**) Mitochondrial transmembrane potential (ΔΨ*m*) detected by JC-1 fluorescence staining in H9C2 cells. Mean fluorescence intensity is displayed on the right of the graphs (n = 6). ^#^ P < 0.05 vs. Control, ^##^ P < 0.01 vs. Control, ^*^ P < 0.05 vs. H_2_O_2_, ^**^ P < 0.01 vs. H_2_O_2_, ^$^ P < 0.05 vs. H_2_O_2_ + SPD, ^$$^ P < 0.01 vs. H_2_O_2_ + SPD. For in vivo studies, the rats were divided into five groups: 1) young (3 months old), 2) old (24 months old), 3) SPD (24-months-old rats treated by SPD for 6 weeks), 4) DFMO (24-month-old rats treated with SPD and DFMO plus MGBG), and 5) EX527 (24-month-old rats treated with SPD and EX527). (**E**) Cardiac aging evaluated by SA-β-gal staining ex-vivo. (**F**) SA-β-gal staining quantification. Scale bars: 20 μm (n = 6). (**G**) Mitochondrial DNA (mtDNA) copy number detected by real-time PCR (n = 8). (**H**) Mitochondrial oxidative phosphorylation (OXPHOS) efficiency was evaluated based on mitochondrial State 3 and 4 oxygen consumption (a), respiratory control rate (RCR) (b), P/O ratio (c), and proton leakage (d); n = 8. ^#^ P < 0.05 vs. young, ^##^ P < 0.01 vs. young, ^*^ P < 0.05 vs. old, ^**^ P < 0.01 vs. old, ^$^ P < 0.05 vs. SPD, ^$$^ P < 0.01 vs. SPD. n = 6 for each group.

To confirm that SPD-induced mitochondrial biogenesis and functional improvement is mediated by SIRT1 activation, **w**e examined the alterations in SIRT1/PGC-1α signaling resulting from the depletion of the polyamine pool through exposure to a combination of difluoromethylornithine (DFMO) and mitoguazone (MGBG), to induce polyamine synthesis inhibition, or to the SIRT1 inhibitor EX-527. As shown in Figure 5A, SPD enhanced the expression of SIRT1, PGC-1α, NRF1, NRF2, and TFAM in NRCMs pre-treated with H_2_O_2_. Meanwhile, both DFMO and EX527 abolished the SPD-mediated increase in protein expression. We next measured the expression of mitochondrial oxidative phosphorylation (OXPHOS) complex I, II, and III subunits, and ATP production in NRCMs (Figure 5B and 5C) and analyzed Δψm changes in H9C2 cells (Figure 5D). We noted that SPD treatment reversed the H_2_O_2_-induced decrease in OXPHOS complex protein expression, ATP levels, and Δψm, whereas DFMO and EX527 partly abrogated these effects.

In in vivo studies, we further observed that depletion of the polyamine pool and inhibition of SIRT1 activity independently increased SA-β-gal-positive staining area (Figure 5E and 5F) and reduced mitochondrial DNA (mtDNA) copy number (Figure 5G) in the myocardium of aged rats treated with SPD. Moreover, SPD-induced improvement in cardiac mitochondrial respiratory function was also remarkably reduced, as indicated by a decrease in mitochondrial State 3 respiration (Figure 5H-a), RCR (Figure 5H-b), and the P/O ratio (Figure 5H-c), and an increase in proton leakage (Figure 5H-d). These results strongly suggest that SPD attenuates cardiac aging by activating SIRT1 and enhancing mitochondrial biogenesis, which leads to improved mitochondrial function in cardiomyocytes.

### SIRT1 mediates spermidine-induced mitochondrial biogenesis through deacetylation and nuclear translocation of PGC-1α

**Figure 6:**
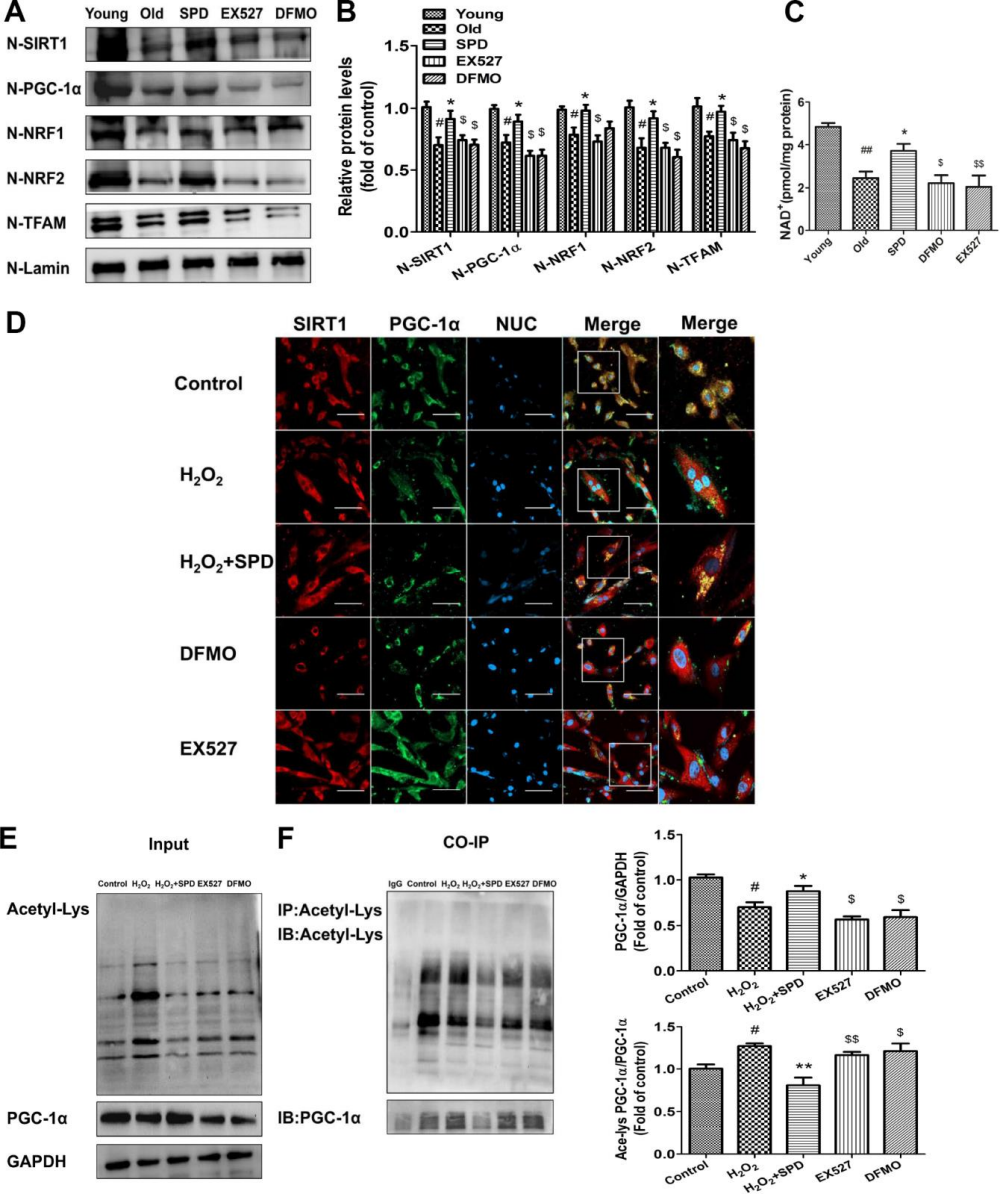
**SIRT1 is required for SPD-induced PGC-1α activation in aging cardiomyocytes.** (**A**) Western blot detection of SIRT1, PGC-1α, NRF1, NRF2, and TFAM in nuclear fractions isolated from cardiac tissue. (**B**) Quantification of protein expression based on L-lamin as a nuclear loading control (n = 4). (**C**) Quantification of NAD+ levels by fluorimetry (n = 8). ^#^ P < 0.05 vs. young, ^##^ P < 0.01 vs. young, ^*^ P < 0.05 vs. old, ^**^ P < 0.01 vs. old, ^$^ P < 0.05 vs. SPD, ^$$^ P < 0.01 vs. SPD. (**D**) Co-localization of SIRT1 (red) and PGC-1α (green) by immunofluorescence in H9C2 cells. Nuclei were stained with DAPI (blue) (n = 8); scale bars: 20 μm. (**E**, **F**) Western blot and immunoprecipitation (IP) analysis of PGC-1α acetylation status in NRCMs. Quantification is shown on the right-hand side of the graphs. GAPDH was used as loading control (n = 4). ^#^ P < 0.05 vs. Control, ^##^ P < 0.01 vs. Control, ^*^ P < 0.05 vs. H_2_O_2_, ^**^ P < 0.01 vs. H_2_O_2_, ^$^ P < 0.05 vs. H_2_O_2_ + SPD, ^$$^ P < 0.01 vs. H_2_O_2_ + SPD.

To verify that SPD-induced upregulation of SIRT1 results in deacetylation and nuclear translocation of PGC-1α, the SIRT1-selective inhibitor EX527 and the inhibitors of polyamine biogenesis were tested in vivo and vitro. Nuclear expression of SIRT1/PGC-1α signaling proteins was significantly decreased in cardiac tissue from aged rats, and these changes were partially reversed by SPD supplementation. In turn, depletion of the polyamine pool and inhibition of SIRT1 activity hampered the upregulation of SIRT1, PGC-1α, NRF1, NRF2, and TFAM induced by SPD (Figure 6A and 6B). Since SIRT1 deacetylase activity is nicotinamide adenine dinucleotide (NAD+)-dependent, we also measured changes in NAD+ content. Consistent with SIRT1 expression data, significantly decreased NAD+ levels were observed in the aged myocardium, compared with young controls. SPD supplementation increased cardiac NAD+ contents in aged rat hearts, and this effect was abrogated by both polyamine depletion and EX527 (Figure 6C).

Next, in vitro studies were performed on H9C2 cells and NRCMs. Using immunofluorescence, we determined that co-localization of PGC-1α and SIRT1 was significantly reduced after exposing H9C2 cells to H_2_O_2_. Exposure to SPD prevented this effect, but failed to do so in the presence of DFMO or EX527 (Figure 6D). Acetylated PGC-1α levels were next measured by western blot and co-IP in NRCMs (Figure 6E and 6F). Compared with control cells, H_2_O_2_ treatment significantly increased the acetylation status of PGC-1α, while SPD treatment had the opposite effect. By contrast, both DFMO and EX527 exposure increased the acetylation status of PGC-1α. Taken together, these data demonstrate that SPD stimulates mitochondrial biogenesis in senescent cardiomyocytes by preserving cellular NAD+ levels and inducing SIRT1-mediated deacetylation of PGC-1α.

### Spermidine stimulates mitochondrial biogenesis through PGC-1α

**Figure 7:**
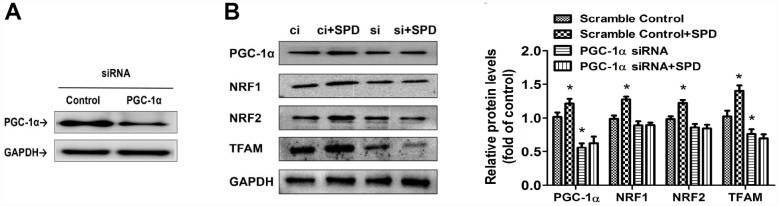
**Deletion of PGC-1α abrogates SPD-mediated enhancement of mitochondrial biogenesis.** (**A**) Western blot analysis of PGC-1α expression in H9C2 cells transfected with siRNA against PGC-1α (si) or control (scrambled) siRNA (ci). (**B**) Western blot and quantification analysis of PGC-1α, NRF1, NRF2, and TFAM expression in transfected H9C2 cells in the absence (ci and si) or presence of SPD (ci + SPD and si + SPD). GAPDH served as loading control (n = 4). ^*^ P < 0.05 vs. Scramble Control.

To further assess the involvement of PGC-1α in the stimulation of mitochondrial biogenesis mediated by SPD, siRNA-mediated PGC-1α knockdown was performed in H9C2 cells (Figure 7A). Following transfection with scrambled control (ci) or PGC-1α-targeted siRNAs (si) for 6 h, cells of the two groups were cultured with SPD for 48 h. Western blot analyses showed that, as expected, SPD exposure significantly upregulated the expression of PGC-1α, NRF1, NRF2, and TFAM in ci-transfected cells. PGC-1α siRNA significantly down-regulated the expression of PGC-1α and TFAM. However, the expression of these proteins was not altered, in SPD-treated cells after PGC-1α knockdown (Figure 7B). These results strongly suggest that SPD promotes mitochondrial biogenesis through PGC-1α.

## DISCUSSION

Aging is an unmodifiable risk factor for heart disease, linked to impaired mitochondrial genesis and function. This increases oxidative stress due to excessive ROS production [1–3], which further impairs mitochondrial respiration, reduces ATP synthesis, and favors mPTP opening and eventually cell death [39, 40]. Therefore, mitochondria-targeting strategies to limit cardiac dysfunction are highly relevant to protect older people against heart disease.

Polyamines are involved in a wide range of cellular processes, including autophagic mitochondrial quality control, anti-inflammatory responses, and protection against oxidative stress [28–34]. Previous studies showed that altered polyamine levels in tissue and blood are associated with aging and various health conditions, including inflammation, diabetes, and neurological disorders [41]. SSAT overactivation was shown to reduce intracellular polyamine stores and catalyze the production of H_2_O_2_ and 3-aminoacetaldehyde, leading to oxidative stress and myocardial damage [42]. Our present study verified that polyamine metabolism was impaired in aged rat hearts. This was evidenced by decreased levels of SP and SPD, reduced expression of ODC, and increased SSAT expression. Noteworthy, exogenous SPD administration replenished cardiac polyamine pools in aged rats. Exogenous SPD supplementation effectively inhibited the expression of senescence marker proteins p21 and p16, preserved normal myocardium ultrastructure, and mitigated mitochondrial damage in cardiac tissue from 24-month-old rats. Additional in vitro experiments in NRCMs and H9C2 cells prematurely aged by exposure to H_2_O_2_ showed that SPD supplementation prevented SA-β-gal staining, consistent with inhibition of cellular senescence in vivo. Due to their good beating function and physiological similarity in situ heart, the NRCMs have become the preferred cell model for studying cardiac pathophysiology in vitro [43]. Despite their embryonic ventricular origin, H9C2 cells do not exhibit spontaneous beating in culture, but show similar energy metabolism and have almost identical hypertrophic responses as NRCMs [44, 45]. Both cell types have been successfully used as an in vitro model for studies of myocardial aging [43, 46]. Therefore, here, the restoration of cardiac polyamine levels observed upon exogenous SPD supplementation is expected to reinstate the protective effects of polyamines on myocardial tissue, and to attenuate or prevent age-related cardiomyocyte dysfunction.

We also found that SPD administration in vivo increased the activity of the antioxidant enzymes SOD and CAT, and improved mitochondrial respiratory activity in the myocardium. Consistent with previous studies on mitochondria from different cell sources [47–49], SPD supplementation inhibited H_2_O_2_-induced ROS accumulation and prevented the decrease in ΔΨm and ATP levels in NRMCs and H9C2 cells. These data suggest that replenishing SPD contents increases antioxidation mechanisms, maintains mitochondrial homeostasis, and delays cardiac aging in the rat.

Accumulating evidence suggests that SIRT1 can promote mitochondrial biogenesis and function via deacetylation of PGC-1α. As a key regulator of mitochondrial biogenesis, PGC-1α controls mitochondrial- and nuclear-encoded mitochondrial gene expression, modulating the transcription of NRF1, NRF2, and TFAM [50]. Sustained mitochondrial biogenesis results in the maintenance of a functional mitochondrial population and an adequate expression of mitochondrial proteins related to the tricarboxylic acid cycle and OXPHOS, contributing to extended life span [19]. In this study, we found that the expression of SIRT1, PGC-1α, NRF1, NRF2, and TFAM in rat cardiac tissue decreased in an age-dependent manner, and correlated differentially with the expression of polyamine metabolism enzymes. Thus, a positive correlation between ODC and both SIRT1 and NRF1, and a negative correlation between SSAT and PGC-1α, NRF1, NRF2, and TFAM became apparent with increasing age. SPD administration to older rats prevented downregulation of SIRT1/PGC-1α signaling proteins in cardiac tissue, while stimulatory effects on SIRT1/PGC-1α pathway expression were further seen in senescent (H_2_O_2_-treated) NRCMs. Since the effects of SPD on SIRT1/PGC-1*α* axis proteins were abrogated by independent inhibition of polyamine synthesis (with DFMO/MGBG) and SIRT1 activity (with EX527), our data suggest a strong link between cardiac polyamine metabolism and mitochondrial biogenesis mediated by the SIRT1/PGC-1α pathway, with important potential implications for cardiac aging.

Disruption of mitochondrial biogenesis slows the organelles’ turnover and aggravates aging by accelerating ROS accumulation, impairing OXPHOS activity, and triggering oxidative damage on lipids, proteins, and DNA [51]. We found that SPD promoted OXPHOS, prevented Δψm decay, and preserved ATP levels in senescent cardiomyocytes in vitro, and these effects were independently abrogated by DFMO and EX527. Furthermore, inhibition of either polyamine biogenesis or SIRT1 activity abrogated the SPD-mediated increase in mtDNA copy number, as well the improvement in mitochondrial respiratory function in aged hearts. Thus, our results suggest that SIRT1 is an essential intermediate in the mechanism by which SPD stimulates mitochondrial biogenesis and function in cardiac cells.

SIRT1 has been shown to increase the transcriptional activity of PGC-1α by inducing its nuclear localization and subsequent deacetylation in an NAD+-dependent manner [52–54], and this was linked to improved metabolic regulation and resistance to oxidative stress [55]. Indeed, overexpression of SIRT1 and subsequent activation of PGC-1α have been associated with a range of health benefits, including protection from metabolic decline and cardiovascular disease [20–22]. We observed that cardiac tissue from older rats given SPD exhibited increased nuclear expression of SIRT1, PGC-1α, and downstream proteins (NRF1, NRF2, and TFAM), and these changes were again prevented by inhibition of polyamine synthesis and SIRT1 activity. Furthermore, our assays showed a similar trend for the changes in myocardial NAD+ levels and the variations in nuclear expression of SIRT1. We observed only weak co-localization of SIRT1 and PGC-1α in H9C2 cells treated with H_2_O_2_. In this culture condition, SPD supplementation remarkably increased SIRT1-PGC-1α co-localization, and this effect was almost abolished by co-exposure to DFMO or EX527. Notably, SPD treatment reduced H_2_O_2_-induced PGC-1α acetylation in NRCMs, which was also promoted by EX527 or polyamine synthesis inhibition, as shown by co-IP. In addition, we showed that PGC-1α deficiency (induced by siRNA) in H9C2 cells partially blocked the expression of NRF1, NRF2, and TFAM. Several new findings have been made about the molecular mechanisms mediating polyamine actions in health and disease [56]. SPD extends life span by triggering deacetylation of histone H3 through inhibition of histone acetyltransferases, suppressing oxidative stress and necrosis [57]. One of SPD’s known targets, the E1A binding protein p300 (EP300) acetyltransferase, regulates forkhead box (FOXO) activity-induced autophagy [58]. In contrast, SPD was proposed to prolong life span and prevent liver fibrosis by inhibiting the deacetylation of MAP1S to enhance autophagy flux [59]. Thus, the seemingly ambiguous effects of SPD on deacetylation and acetylation reactions, and its precise role on life span regulation, remain an important, unsolved problem in the field of molecular cell biology.

Worth stressing, our present data provide novel evidence that SPD enhances mitochondrial biogenesis by increasing the nuclear expression of PGC-1α, which is mediated by enhancing the NAD+-dependent deacetylase activity of SIRT1 in aging rat hearts. Polyamines exist in mammalian cells mainly as RNA-polyamine complexes. By microarray analysis of human hair follicle mRNA extracts, Ramot et al. revealed that SPD can upregulate several key target genes implicated in the control of mitochondrial function [60]. We recently demonstrated that SPD can increase the expression of PGC-1α mRNA in the newborn offspring myocardium exposed to maternal hypoxia [61]. Therefore, the mechanisms underlying SPD-induced mRNA expression changes in signaling molecules of the SIRT1/PGC-1α pathway in the aging myocardium deserve further exploration.

In conclusion, we provided evidence that mitochondrial biogenesis and function are compromised in cardiac tissue from aged rats, in parallel with age-dependent depletion of polyamine stores and downregulation of signaling molecules within the SIRT1/PGC-1α pathway. Through ex-vivo and in vitro analyses, we determined that SPD ameliorated cardiomyocyte aging by activating the SIRT1/PGC-1α signaling pathway, enhancing in turn mitochondrial biogenesis and function. These findings might guide new therapeutic strategies for counteracting cardiac aging and preventing age-related cardiovascular diseases, and lay a foundation for improving the treatment of heart diseases related to mitochondrial dysfunction.

## MATERIALS AND METHODS

### Animal experiments

Three-month-old and 24-month-old male Wistar rats were purchased from the experimental animal centre of Harbin Medical University. The animals were acclimated for 4 weeks in a quiet environment, at room temperature (25 °C) with 50% humidity, a 12 h/12 h light/dark cycle, and standard rodent chow and water provided *ad libitum*. Subsequently, rats were randomly separated into five groups: (1) Control: 3-month-old rats intraperitoneally injected with normal saline for 6 weeks; (2) Old: 24-month-old rats intraperitoneally injected with normal saline for 6 weeks; (3) SPD: 24-month-old rats intraperitoneally injected with SPD (Sigma, St. Louis, MO, USA) dissolved in normal saline (10 mg/kg/d) for 6 weeks before sacrifice; (4) DFMO, 24-month-old rats were given SPD for 6 weeks and simultaneously provide with 2% α-difluoromethylornithine (DFMO; Sigma, St. Louis, MO, USA) in drinking water and intraperitoneally infused with mitoguazone (MGBG, Sigma, St. Louis, MO, USA) dissolved in normal saline (15 mg/kg/d) in the last week; and (5) EX527, 24-month-old rats were administered SPD for 6 weeks and simultaneously provide with EX527 intraperitoneally (a SIRT1 inhibitor; Selleck, Shanghai, China) at 5 mg/kg/d in the last week.

### Transmission electron microscopy and mitochondrial area measurement

A portion of the left ventricular apex, approximately 3.0 × 4.0 × 1.0 mm^3^ in size, was placed in glutaraldehyde phosphate buffer for 30 min and then cut into small pieces of approximately 1~2 mm^3^ to continue fixation for 24 h at 4 °C. After conventional dehydration, soaking, embedding, and dyeing procedures, 50-70 nm ultrathin slices were obtained. Myocardial ultrastructure was observed using a transmission electron microscope (TEM; S4800 Hitachi, Tokyo, Japan). Mitochondrial area measurement was performed as previously described [62]. Briefly, two left ventricle tissue pieces from each rat were randomly chosen. Images were acquired at a magnification of 10,000× when an area containing longitudinal myofilaments surrounded by a mitochondrial network was observed. Individual mitochondria and myofilaments were delineated, and mitochondrial areas were quantified using Image J software.

### Determination of polyamine content

Extraction and benzoylation of polyamines from the left ventricular tissue of rats was based on our previously published methods [37]. Polyamine content was measured using high-performance liquid chromatography (HPLC; Waters Co., Milford, USA). Polyamine derivatives were separated on a Hypersil ODS C18 column (250×4.6 mm, 5 μm; Waters Co.), eluted with methanol and distilled water (v/v, 65/35) at 30 °C, and monitored using an ultraviolet detector at 229 nm. The polyamine concentration was expressed in nanomoles of amines per gram of wet tissue (nmol/g w.w.).

### Determination of senescence-associated β-galactosidase activity

Senescence-associated β-galactosidase (SA-β-gal) staining was performed according to the instructions of the SA-β-galactosidase Staining Kit (Beyotime Inc., Haimen, China C0602). In brief, cryostat sections were fixed in 3% formaldehyde for 15 min, followed by three washes in phosphate-buffered saline (PBS) at room temperature. Slides were immersed in freshly prepared SA-β-gal staining solution and then incubated at 37 °C overnight. Stained sections were washed twice with PBS and counterstained for 1 min with hematoxylin. Samples were analyzed under an optical microscope by two independent investigators in a blind fashion. All samples were stained in triplicate. The activity of the senescence marker was scored as the percentage of dark blue areas. SA-β-gal staining was also performed, following protocol instructions, on neonatal rat cardiomyocytes and H9C2 cells.

### Measurement of antioxidant enzyme activity

Superoxide dismutase (SOD) and catalase (CAT) activities in cardiac tissue were measured using commercial kits (SOD: A001-3-1; CAT: A007-2-1; Jiancheng Bio. Institute, Nanjing, China) with a spectrophotometer (Perkin- Elmer, Norwalk, CT, USA). Protein concentration was measured using the bicinchoninic acid method (Pierce, Rockford, USA) with bovine serum albumin (BSA) as a standard.

### Mitochondrial isolation

Mitochondria were extracted from cardiac tissue using a Tissue Mitochondria Isolation Kit (Beyotime Biotechnology, Shanghai, China C3606). Briefly, after treatment, rats’ ventricular muscle tissues were cut and weighed. Samples were cut into smaller pieces and digested with trypsin by adding the appropriate volume of mitochondrial separation reagent. Each sample was ground 20-30 times in a Dounce homogenizer and centrifuged at 600 g for 5 min at 4 °C. Thereafter, the supernatant was transferred and centrifuged at 11,000 g for 10 min at 4 °C. Pelleted mitochondria were divided and one fraction preserved in Mitochondria Storage Buffer (Beyotime Biotechnology, Shanghai, China C3609) for immediate detection of respiratory rate, while the other was stored at −80 °C for western blot analysis.

### Determination of mitochondrial respiratory function

State 3 and State 4 oxygen consumption rates, respiratory control rate (RCR), and ADP consumed/oxygen consumed (P/O ratio) with pyruvate and malic acid as substrates were measured in isolated myocardial mitochondria using a Clark oxygen electrode (Hansatech Instruments, Norfolk, UK) at 30 °C in mitochondrial respiration buffer as previously described [36]. When the oxygen electrode reaction chamber temperature was 25 °C, 500 μg of mitochondrial protein was added to the buffer solution. After equilibration for 1 min, 5 mmol/L pyruvate and 5 mmol/L malate were added to the buffer to initiate the respiration test. State 3 respiration begun after addition of 0.2 μmol/L of ADP; once ADP was exhausted, State 4 respiration ensued. RCR, an important index to evaluate mitochondrial respiratory function, reflects mitochondrial oxidative phosphorylation (OXPHOS) coupling efficiency and is expressed by the ratio of the State 3/State 4 oxygen consumption rates, representing ADP availability and depletion, respectively. P/O in turn is the ratio of the number of moles of ADP added to the reaction system to the number of moles of oxygen consumed, which represents the efficiency of mitochondrial OXPHOS.

### Mitochondrial DNA copy number estimation

Cardiac tissue mtDNA was extracted using a whole genome DNA extraction kit (Zoman Biotechnology, Beijing, China), and its concentration measured on a Nanodrop 2000 instrument. Real-time PCR was conducted with a 2x SYBR Green qPCR Master Mix Kit in a LightCycler 96 real-time PCR cycler. The total PCR volume was 20 μl, with 1 μL each of 10 μmol/L upstream and downstream primers, 1 μl of cDNA, 7 μL of SYBR Green qPCR Master Mix, and 10 μL of deionized water. Two-step amplification involved activation of the Hot-Start DNA Polymerase for 10 min at 95 °C. PCR conditions involved 40 cycles, with each cycle consisting of denaturing at 95 °C for 15 s, extension at 60 °C for 60 s and melting at 95 °C for 15 s, 60 °C for 60 s, and 95 °C for 15 s. The primers were as follows: Cytochrome B: Forward: 5′-GCAGCTTAACATTCCGCCCAATCA-3′; Reverse: 5'-TGTTCTACTGGTTGGCCTCCGATT-3′; Actin: Forward, 5'-TCGTGCGTGACATTAAAGAG-3′; Reverse: 5'-ATTGCCGATAGTGATGACCT-3′; Calculation formula: ^Δ^CT=(β-actin CT)-(Cyt B CT), mtDNA copy number=2×2^Δ^CT.

### Nuclear isolation

Nuclear and cytosolic protein fractions were extracted from cardiac tissue using the Nuclear and Cytoplasmic Protein Extraction Kit (P0027; Beyotime Biotechnology, Shanghai, China). Briefly, tissue samples were cut into pieces and homogenized in the presence of phenylmethylsulfonyl fluoride (PMSF). Each mixture was then placed in an ice bath for 15 min and then centrifuged at 1500 g for 15 min at 4 ºC to separate cytoplasmic proteins (supernatant) and cell nuclei (precipitate). Corresponding extraction reagents were next added to each fraction following the manufacturer’s protocol. The samples were stored at −80 °C for subsequent western blot analysis.

### Nicotinamide adenine dinucleotide (NAD+) measurement

NAD+ from cardiac tissue and cultured cells was estimated colorimetrically with a NAD/NADH assay kit (ab65348; Abcam, Cambridge, MA, USA) according to the manufacturer’s instructions.

### Cell culture

Primary neonatal rat cardiomyocytes (NRCMs) were obtained from Wistar rats aged 1-3 days as previously described [63]. All experiments were approved by the Animal Care Committee for the Use of Experimental Animals at Harbin Medical University (Heilongjiang, China). Briefly, the hearts were cut into pieces, digested with trypsin for 8 minutes and terminated digestion with DMEM culture solution containing 10% fetal calf serum. After 7 times of digestion, the cells were collected by centrifugation at 600 g for 10 min at 4 °C. Two hours after incubation at 37 °C/5% CO_2_, the attached cells were discarded, and unattached cardiomyocytes were re-cultured in collagen-coated 35 mm Petri dishes or 8-, 24- or 96-well plates in DMEM containing 10% fetal bovine serum (FBS) and 1% penicillin or streptomycin, in a humidified incubator at 37 °C/5% CO_2_. The media was changed twice a week. The H9C2 cell line (purchased from the Chinese Academy of Sciences in Shanghai, China) was cultured in DMEM containing 10% FBS and 1% penicillin or streptomycin in an incubator at 37 °C with 5% CO_2_.

### Cell viability assays

For cell viability assessment, NRCMs were cultured for 48 h in 96-well plates and then exposed to 40 μmol/l H_2_O_2_ over 4 h (see "Supplementary Materials"). The medium was then replaced with normal medium, and various concentrations of SPD (0-100 μmol/L) were added for 48 h. Alternatively, following exposure to H_2_O_2_, 10 μmol/L SPD was added to fresh culture medium and NRCMs incubated for different time periods up to 48 h. Cell viability was determined using the Cell Counting Kit-8 (Solarbio, Beijing, China CA1210) according to manufacturer's instructions. Absorbance (450 nm) was measured, and results expressed as percentages relative to control (100%).

### Cell experiments

NRCMs and H9C2 cells were randomly divided into five experimental groups: (1) Control: high glucose-DMEM with 10% FBS; (2) H_2_O_2_: 40 μmol/l H_2_O_2_ for 4 h, and incubation in normal medium for additional 48 h; (3) H_2_O_2_-SPD: 40 μmol/l H_2_O_2_ for 4 h, and incubation in normal medium with 10 μM SPD for additional 48 h; (4) H_2_O_2_+SPD+DFMO (DFMO): 2 mmol/L DFMO plus SPD for 48 h followed by H_2_O_2_ for 4 h; (5) H_2_O_2_+SPD+EX527 (EX527): 40 μM EX527 plus SPD for 48 h followed by H_2_O_2_ for 4h. Aminoguanidine (1 mM) was added to the medium 5 min before SPD treatment to inhibit the activity of PAO in FBS.

### Cellular ATP measurement

ATP concentration in primary cultured NRCMs was assayed using an ATP Assay Kit (S0027; Beyotime Biotechnology, Shanghai, China) according to the manufacturer’s instructions and measured on a Luminoskan™ Ascent luminometer (Thermo Fisher Scientific Inc., USA). An ATP calibration curve was used for data normalization.

### Mitochondrial transmembrane potential and ROS production assays

To evaluate mitochondrial transmembrane potential (Δψm), the cells were stained with tetramethylrhodamine ethyl ester (TMRE, AAT Bioquest Inc. Sunnyvale, CA, USA) for 20 min at 37 °C, or with JC-1 (Mitochondrial Membrane Potential Assay Kit; C2006, Beyotime Inc., Shanghai, China). ROS production in living cells was assessed by dihydroethidium (DHE) staining (S0063, Beyotime Inc., Shanghai, China). To detect the production of mitochondrial superoxide, the cells were stained with MitoSOX (M36008, ThermoFisher Scientific, Waltham, MA, USA). Images were taken with an Olympus FluoView FV1000 fluorescence microscope (Olympus Optical Co., Ltd., Takachiho, Japan). Results were compiled from 20–45 cells from three or more independent experiments, and the same microscope settings were used for all treatments.

### Western blot analysis

Frozen left ventricular cardiac tissue and isolated nuclear samples were homogenized in ice-cold RIPA lysis buffer (P0013B; Beyotime Inc., Shanghai, China) with 50 μg/ml PMSF and incubated on ice for 40 min. Homogenates were next centrifuged at 10,000 g for 15 min at 4 °C to remove cellular debris and isolate total protein. Cardiomyocytes were harvested and lysed in the same RIPA buffer containing PMSF. Protein concentration was determined using a Bradford assay kit (P0006C; Beyotime Inc., Shanghai, China). Equal amounts of protein from different experimental groups were loaded and separated on 10% SDS-PAGE gels, and electrophoretically transferred onto 0.2 μm PVDF membranes. The membranes were blocked in TBS-T containing 5% (w/v) skim milk at 37 °C for 1 h, and incubated overnight at 4 °C with primary rabbit antibodies specific to GAPDH (10494-1-AP), p21 (10355-1-AP), p16 (10883-1-AP), SOD (24127-1-AP), CAT (21260-1-AP), GLB1 (15518-1-AP), SIRT1 (13161-AP), TFAM (19998-1-AP), NRF1 (12482-1-AP), NRF2 (16396-1-AP), SDHB (10620-1-AP), NDUFV2 (15301-AP), or UQCRC (14742-1-AP). All polyclonal antibodies were from Proteintech (Wuhan Sanying Biotechnology, China). Antibodies against ODC (sc-33539) and SSAT (sc-67159) were from Santa Cruz Biotechnology (Dallas, TX, USA). Goat anti-PGC-1α (ab106814) was from Abcam (Cambridge, MA, USA), and mouse anti-lamin B1 (66095-1-Ig) was from Proteintech (China). Horseradish peroxidase (HRP)-conjugated secondary antibodies (ZB-2301, ZB-2305, ZB-2306; Zhongshan Golden Bridge Biotechnology, Beijing, China) were used to label primary antibodies. Immunoreactive proteins were then developed using ultrasensitive ECL luminescent solution (Proteintech), quantified using a FluorChem Chemiluminescence Imaging System (Protein Simple, San Jose, CA) via densitometry, and normalized to that of GAPDH. The final results were expressed as relative protein levels by normalizing the data to control values.

### Immunofluorescence

Following treatment with SPD, H9C2 cells were seeded onto glass cover slips in 24-well plates. For detection of PGC-1α and SIRT1, cells were fixed in 4% paraformaldehyde for 15 min, permeabilized with PBS/0.1% Triton X-100, blocked with 3% BSA in PBS, and incubated with PGC-1α (sc-518038) and SIRT1 (sc-15404) antibodies (Santa Cruz Biotechnology, Dallas, TX, USA) overnight at 4 °C. Fluorescent secondary antibodies (ab150113 and ab150075; Abcam, Cambridge, MA, USA) were used for detection according to the manufacturer’s instructions. Immunofluorescence was assessed with a confocal microscope (Carl Zeiss LSM510, Tokyo, Japan). Confocal laser scanning microscopy analysis of PGC-1α and SIRT1 colocalization was performed for H9C2 cells.

### Detection of protein acetylation through co-immunoprecipitation

To detect PGC-1α acetylation in cells, cultured cardiomyocytes were collected and lysed in lysis buffer (P0013C; Beyotime, Shanghai, China) for 30 min at 4 °C. After centrifugation at 14,000 g for 20 min at 4 °C, the supernatants were incubated with 1 μg of anti-PGC-1α antibody overnight at 4 °C. Rabbit IgG (Santa Cruz Biotechnology) was used as control. Each sample was incubated with protein A/G magnetic bead slurry (Selleckchem, Houston, TX, USA) for 2 h at 4 °C to avoid nonspecific binding. A total of 500 μg of protein from each sample was incubated with 2 μg of specific antibody to acetylated lysine (#9441, Cell Signaling Technology, Danvers, MA, USA) overnight at 4 °C according to the instructions of the Protein A/G Magnetic Beads for IP. The blot was probed with rabbit anti-PGC-1α (Abcam) and anti-acetylated lysine antibodies and then treated with appropriate secondary antibodies conjugated to HRP (Santa Cruz Biotechnology, Dallas, TX, USA). Then, western blotting was performed as described above.

### siRNA-mediated PGC-1α knockdown

For short interfering RNA (siRNA)-mediated knockdown studies, H9C2 cells were seeded into six-well plates 48 h prior to transfection on serum-free DMEM. Cells were transfected with scrambled, nonspecific siRNA or siRNA targeting the mouse PGC-1α gene (GenePharma, Shanghai, China) using Lipofectamine 2000 Reagent (Invitrogen, Carlsbad, CA, USA) for 6 h according to the manufacturer's instructions. Next, H9C2 cells were treated with SPD for 48 h for subsequent assays.

### Statistical analyses

All statistical analyses were performed using SPSS 17.0 (SPSS. Chicago, IL, USA). Values are expressed as mean ± SD. The t-test was used for comparison between two groups. Comparisons between the mean of three or more groups were done by one-way ANOVA. Correlation analysis was performed by linear regression. Differences were considered significant at p < 0.05. Each experiment consisted of at least three replicates per condition.

## Supplementary Material

Supplementary Materials

Supplementary Figures
