# Supplementary material for: Spermidine alleviates cardiac aging by improving mitochondrial biogenesis and function
Source: Aging (Albany NY). 2020 Jan 6;12(1):650–71. doi: 10.18632/aging.102647 (PMC6977682; doi:10.18632/aging.102647)
Supplement: Supplementary Materials [file aging-12-102647-s001..pdf]

SUPPLEMENTARY MATERIALS

Effects of H_2O_2 on cell viability

Primary neonatal rat cardiomyocytes (NRCMs; Supplementary Figure 1) and H9C2 cells (Supplementary Figure 2) were exposed to various concentrations of H_2O_2 for 4 h (left graphs) or to 40 μmol/L H_2O_2 for different times (right graphs), and subsequently cultured for additional 48 h in normal medium. The viability of cardiomyocytes was evaluated using the CCK8 assay. We established an optimal sub-lethal concentration of 40 μmol/L of H_2O_2, and a treatment duration of 4 h to induce premature senescence in cell cultures.
