# Supplementary material for: Spermidine alleviates cardiac aging by improving mitochondrial biogenesis and function
Source: Aging (Albany NY). 2020 Jan 6;12(1):650–71. doi: 10.18632/aging.102647 (PMC6977682; doi:10.18632/aging.102647)
Supplement: Supplementary Figures [file aging-12-102647-s002..pdf]

SUPPLEMENTARY FIGURES

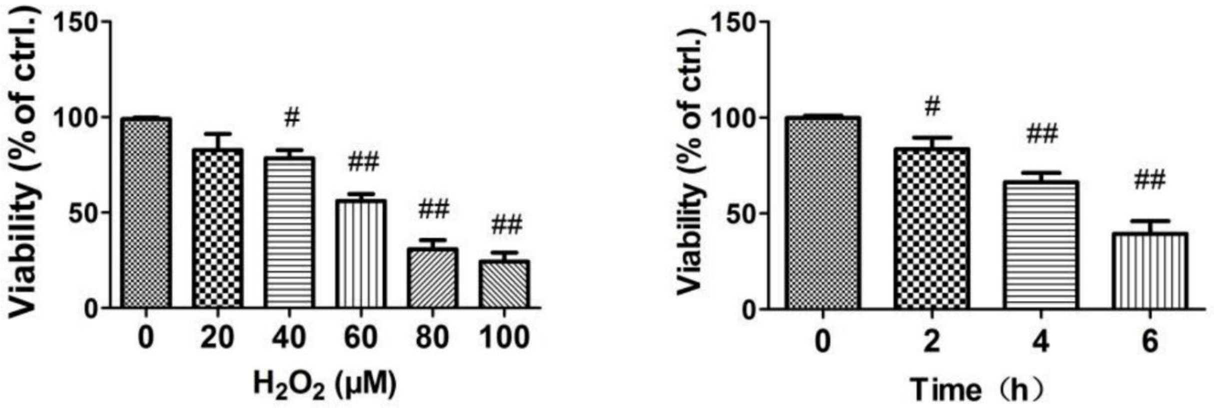

Supplementary Figure 1. Effect of hydrogen peroxide on cell viability of primary NRCMs. Bars = means ± SD; n = 8 per group. $^{\#}$ P < 0.05 vs. blank group. $^{\#\#}$ P < 0.01 vs. blank group.

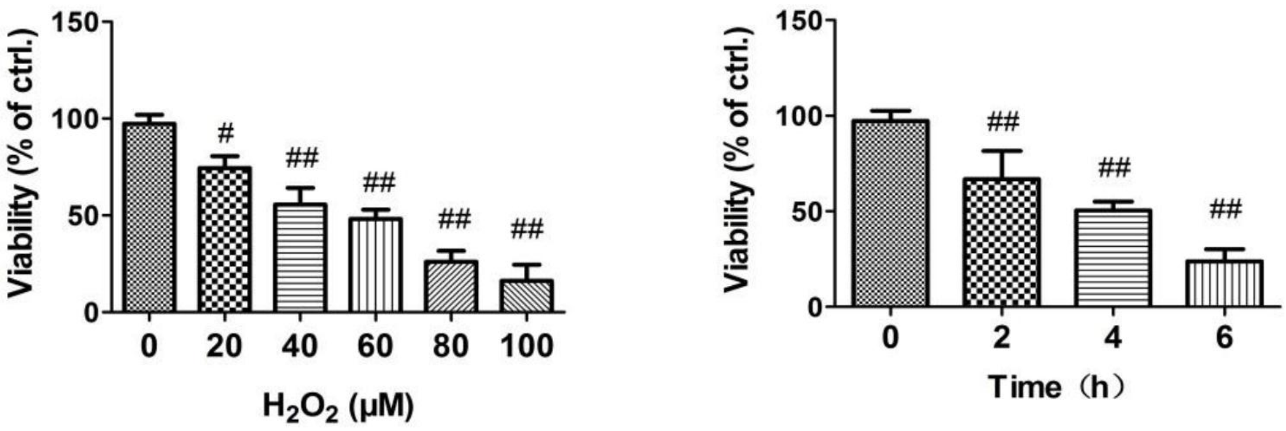

Supplementary Figure 2. Effect of hydrogen peroxide on the viability of H9C2 cells. Bars = means ± SD; n = 8 per group. $^{\#}$ P < 0.05 vs. blank group. $^{\#\#}$ P < 0.01 vs. blank group.
